# Analyzing Foot Posture Malalignments in Obese Individuals

**DOI:** 10.7759/cureus.79373

**Published:** 2025-02-20

**Authors:** Durva Hande, Sawani Aphale, Sandeep Shinde, Manoj P Ambali, Prakash Patil

**Affiliations:** 1 Department of Musculoskeletal Sciences, Krishna College of Physiotherapy, Krishna Vishwa Vidyapeeth, Deemed to be University, Karad, IND; 2 Department of Anatomy, Krishna College of Physiotherapy, Krishna Vishwa Vidyapeeth, Deemed to be University, Karad, IND; 3 Radiodiagnosis, Krishna Institute of Medical Sciences, Karad, IND

**Keywords:** aged, females, flatfoot, gait, obesity

## Abstract

Background and objective

Obesity often leads to foot deformities due to the increased pressure on the feet, the body's most distal parts. Obesity-induced foot malalignments can impact foot posture, making it essential to study these changes for better understanding and management. Hence, the study aimed to examine foot posture malalignments in obese individuals.

Methods

In this cross-sectional study, 216 participants - 108 obese and 108 with normal BMI - were selected based on inclusion and exclusion criteria. The study was performed at Krishna College of Physiotherapy (KCPT), Karad. A well-elaborated assessment was done using Foot Posture Index-6, navicular drop test, degree of toe-out, Meary’s angle (MA), and forefoot and rearfoot deviations. This study was statistically analyzed using SPSS Statistics software (IBM Corp., Armonk, NY), and results were obtained accordingly.

Results

Among 216 participants, regarding the FPI-6 assessment, those in the age group of 43-50 years experienced a significant impact on foot (p=0.0003), particularly among the females (p=0.0002). As for the navicular drop test, the 43-50 age group was significantly affected (p=0.0032), with females again being most affected (p<0.0001). While assessing the toe-out angle, the 43-50 age group was significantly affected (p=0.0007) and females were substantially affected (p=0.0018). For MA, the age group of 35-42 and 43-50 years of obese BMI group were significantly affected, with males and females both affected significantly (p<0.0001). Abnormal rearfoot valgus and forefoot varus were combined and seen in the obese population and had a significant impact on the elderly and females (p<0.0001).

Conclusions

Based on our findings, obese individuals are more susceptible to foot posture malalignments than the normal BMI group. The study also highlighted that the elderly and females are more prone to have altered foot posture, predominantly flat feet.

## Introduction

Globally, more than 1.9 billion adults are classified as overweight, with approximately 650 million categorized as obese [1]. Obesity is an excessive accumulation of adipose tissue that presents significant health risks, typically characterized by a BMI exceeding 30. This condition arises from an imbalance between energy intake and expenditure, accumulating excess body fat. Recent studies have indicated that over 50% of Indian adults experience abdominal obesity, with approximately 12% being classified as obese, and abdominal obesity being more prevalent than other forms across all BMI categories [2]. Individuals with obese BMI are particularly at an elevated risk of developing various chronic diseases [3].

Obesity is a multifactorial condition influenced by a complex interplay of genetic, environmental, and behavioral factors, with lifestyle choices being a primary contributor. Sedentary behavior, poor dietary habits, inadequate sleep, high-stress levels, comorbid health conditions, and genetic predisposition all significantly contribute to the onset and progression of obesity [4]. Furthermore, a clear association exists between obesity and foot-related complications, including pain, disability, and postural alterations. Obesity can affect foot structure through mechanisms such as changes in arch formation, alterations in the plantar fat pad, increased plantar pressure, and modifications in gait patterns [5,6]. These factors collectively contribute to the increased risk of musculoskeletal issues in individuals with obesity.

The arches of the foot are integral to the assessment of foot posture and gait mechanics, serving crucial functions in weight distribution, shock absorption, and adaptation to uneven surfaces. These structures are maintained by a combination of anatomical components, including the osseous structure, ligaments, muscles, and tendons, which collectively act in a manner akin to supportive slings [7]. Three primary foot types are identified: planus, characterized by a low arch accompanied by a valgus hindfoot; rectus, representing a well-aligned hindfoot and forefoot; and cavus, which features a high arch with a varus hindfoot. Individuals with flat feet often exhibit pronation, resulting in a medial shift of ground reaction forces, while those with high arches tend to supinate, directing forces laterally during stance. The condition of flatfoot, or the collapse of the medial longitudinal arch, can manifest in both neutral and weight-bearing postures. In contrast, the high arched feet characterized by the presence of elevated arch are present in all the postures that are in weight-bearing and in nonweight-bearing positions also. The structural integrity of the foot is critical in understanding how body weight is distributed across its various components [8,9].

Foot-related pathologies in obese individuals have been shown to significantly diminish quality of life, with increasing BMI strongly correlating with the onset of foot pain and disability [6]. This association underscores both biomechanical and metabolic mechanisms at play in the development of these conditions. Moreover, plantar pressure has been found to correlate with distinct foot postures, wherein structural alterations to the foot lead to variations in pressure distribution and angulation [10]. The alignment of the foot plays a pivotal role in the progression of knee osteoarthritis (OA), influencing the dynamics of the lower kinetic chain. Abnormal foot mechanics, such as overpronation (excessive inward rolling of the foot) and supination (outward rolling), disrupt the load distribution on the knee joint. This aberrant force distribution, particularly during ambulation or weight-bearing, exacerbates knee OA by increasing localized stress, particularly in the medial compartment, which is more susceptible to degenerative changes. Overpronation results in a medial shift of body weight, promoting a valgus knee position (knock-knee), whereas supination directs weight laterally, potentially inducing a varus knee alignment (bow-legged). Chronic exposure to such altered loading patterns accelerates the progression of OA, leading to heightened pain, diminished mobility, and further joint deterioration [11].

Obesity is closely linked to a low-arched (planus) foot posture that acquired a flat foot which develops over time unlike congenital flatfoot, excessive pronation during dynamic foot function, and elevated plantar pressures during walking. These biomechanical alterations may contribute to gait abnormalities, reduced postural stability, and an increased risk of musculoskeletal complications affecting the lower extremities. Obesity in older adults is closely associated with excessive body mass, diminished physical fitness levels, and an elevated risk of falls. Moreover, structural foot abnormalities in this population have been identified as a significant risk factor for falls. Individuals who experience falls may develop a heightened fear of falling, leading to further restrictions in both physical activity and social engagement. This self-perpetuating cycle ultimately exacerbates obesity and its associated health complications [12].

Excessive body mass is a significant risk factor for the development of flatfoot deformity, which compromises the structural integrity and stability of the hindfoot [13]. Additionally, an elevated BMI has been associated with reduced postural stability during standing, potentially requiring compensatory mechanisms such as increased ankle joint stiffness to maintain equilibrium [13]. Higher BMI is linked to mobility challenges, which may elevate the risk of falls in individuals with obesity [14].

Given that the foot is the most distal part of the human body, it experiences significant pressure and loading. Structural malalignments in the foot can lead to uneven distribution of these forces, contributing to a variety of strains, sprains, and injuries that warrant further investigation. This study could serve as a foundation for exploring preventive strategies and treatment options aimed at improving foot health, which would ultimately enhance overall quality of life.

## Materials and methods

A total of 216 participants were randomly selected for this cross-sectional study, which was rigorously designed to examine foot posture and related factors in individuals classified as obese BMI and individuals with normal BMI. The participants were recruited from a population in Karad, with the inclusion criteria targeting adults aged between 18 and 50 years. The sample comprised 108 females and 108 males, all of whom were categorized as either obese BMI (Grade 1 obese BMI category) for group B and within the normal BMI range for group A. The samples were categorized by using SPSS 26.0 software. This specific focus was intended to assess the potential impact of obesity on foot posture and the associated biomechanical factors. Exclusion criteria were individuals with fractures, open injuries, or other significant musculoskeletal conditions were excluded, as these conditions could have confounded the study’s outcomes. Additionally, participants falling within the overweight BMI category were not included in the study. The selection process also took into account the participants' willingness to engage in the study, with only those who voluntarily agreed to participate being included.

The research adhered to strict ethical standards and received approval from the Institutional Ethical Committee of Krishna Vishwa Vidyapeeth, as indicated by the approval date of 18/10/2023 and the reference number 137/2023-2024. The study ensured the protection of participants’ rights and welfare, in alignment with the ethical guidelines outlined in the Declaration of Helsinki. All participants were thoroughly informed about the study’s objectives, procedures, and potential implications, ensuring transparency and obtaining informed consent. Moreover, the confidentiality of participants was rigorously maintained throughout the study, underscoring the commitment to upholding ethical research practices. Sample size was calculated by using this formula, n = Z^2^ pq/L^2^= 216, where n = sample size, Z = standard normal variant at 95% = 1.96, p = proportion of obese individuals, q = 100 - p, L = 5(permissible limit of error), n = 216

Each participant underwent a detailed assessment of their foot posture, which was evaluated using the following methods: Foot Posture Index-6 (FPI-6), navicular drop test, degree of toe out, Meary’s angle (MA), forefoot, and hindfoot deviations. The study was conducted from 25/10/2024 to 15/12/2024.

Outcome measures

Foot Posture Index (FPI-6)

The FPI is widely recognized as a reliable and comprehensive tool for evaluating foot posture, providing a systematic approach to assessing the alignment and positioning of the foot [10]. This method is grounded in the evaluation of six specific parameters, each of which contributes to a detailed understanding of an individual's foot posture. The FPI is noted for its high intra-rater reliability, with an intraclass correlation coefficient (ICC) of 0.96 and a p-value of less than 0.001, indicating strong consistency in measurements when the same examiner assesses the foot posture multiple times [15]. During the assessment process, the examiner carefully palpates the talar head bilaterally, which is a crucial step in determining the position of this bone in comparison to the rest of the foot.

The other components are the supra and infra malleolar curvature, which refers to the shape and alignment of the areas above and below the ankle bones; the talonavicular prominence, which evaluates the protrusion of the talonavicular joint; and the calcaneal frontal plane position, which assesses the alignment of the heel bone in the frontal plane. Additionally, the FPI includes the assessment of the abduction or adduction of the forefoot about the rearfoot, which provides insight into the rotational alignment of the foot. Finally, the medial longitudinal arch congruence is evaluated to determine the height and shape of the arch, which is an important factor in overall foot biomechanics. By examining all six parameters, the FPI offers a thorough and reliable measure of foot posture, making it an invaluable tool in both clinical and research settings.

Navicular Drop Test

When assessing a participant using the navicular drop test, the examiner begins by palpating the navicular bone, which is a key structure in the foot's medial arch. The initial measurement is taken with the participant in a seated position, where the foot is unloaded, and the examiner carefully measures the vertical distance from the navicular bone to the ground. This provides a baseline reading of the navicular height when the foot is in a non-weight-bearing position. Next, the same measurement is repeated with the participant standing, which shifts the foot into a loaded, weight-bearing position. This allows the examiner to observe how the foot's arch responds to the body's weight, particularly looking for any changes in the position of the navicular bone.

The difference in the measurements taken between the seated and standing positions is then calculated, with this difference being referred to as the navicular drop. The navicular drop is a critical indicator of the amount of foot pronation or the flattening of the medial longitudinal arch that takes place when the participant is standing. A navicular drop greater than one centimeter is generally considered abnormal and suggests excessive pronation that is flatfoot [16]. This can be correlated with various biomechanical issues, such as overpronation, which leads to discomfort or injury. By identifying abnormal navicular drop values, the test provides a better understanding of the participant’s foot mechanics, which can be used to guide further assessment, treatment, or intervention strategies. 

Degree of Toe-Out

The foot angle, also known as the degree of toe-out, is measured as the angle between two lines. The first line represents the direction of movement, while the second line passes through the midpoint of the heel and the second toe. The examiner should note down how the person stands and how the gait of the person is. In a normal person, 50-60% of the weight is usually taken by the heel. To measure this, the subject is instructed to walk forward, and starting from the second footprint, three consecutive footprints are analyzed to determine the toe-out angle. The normal degree of toe out is 120-180 from the body’s sagittal axis, which is 50 in children. Abnormal toe out angle can lead to various gait-related complications and that will change the normal gait pattern in person [17].

Forefoot and Rearfoot Deviations

Forefoot angle (FFA): For all participants, FFAs in both the subtalar joint neutral position and the prone lying position were measured following standard measurement protocols.

Rearfoot angle (RFA): For the measurement of RFA, longitudinal bisection lines were first drawn using a marker along the posterior aspect of the lower third of the leg and the calcaneus to define the bisection of both the lower one-third of the leg and the calcaneus, with the subject positioned in a prone posture. Subsequently, the subject assumed a unilateral stance on a box within their gait template. The relaxed RFA was then measured as the angle between the bisection of the lower one-third of the leg and the bisection of the calcaneus, with the measurement recorded in degrees [18].

Meary’s Angle

MA measures the tilting of both the talus and the first metatarsal bones. It is a radiographic measurement that is frequently used to diagnose conditions like flatfoot or pes planus. If the meary’s angle is between 0 and 4 degrees, the foot is considered to be within the normal alignment. If the angle is greater than 4 degrees but less than 15 degrees, it is classified as a case of flatfoot. Finally, if the angle exceeds this range, the condition is categorized as moderate flatfoot [19].

**Figure 1:**
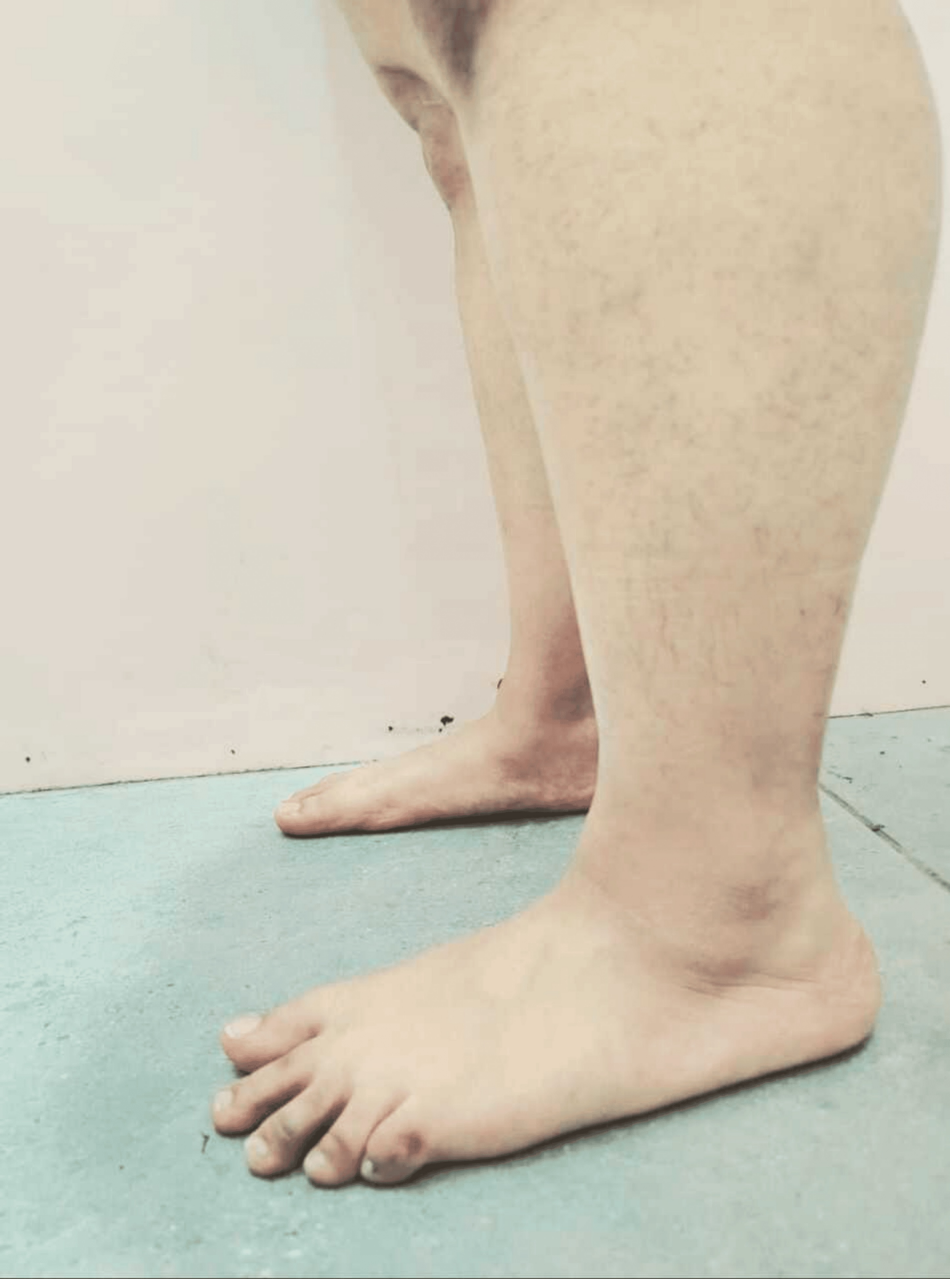
Lateral view of lower limb of a 23-year-old female showcasing the foot posture deformities including hyperextension of knee and flattening of medial longitudinal arch of a right foot

Figure 1 illustrates the lateral malleolus, highlighting its anatomical position on the outer side of the ankle. Additionally, the diagram depicts the lateral arch of the feet, showcasing its structural integrity and alignment in weight-bearing posture.

**Figure 2:**
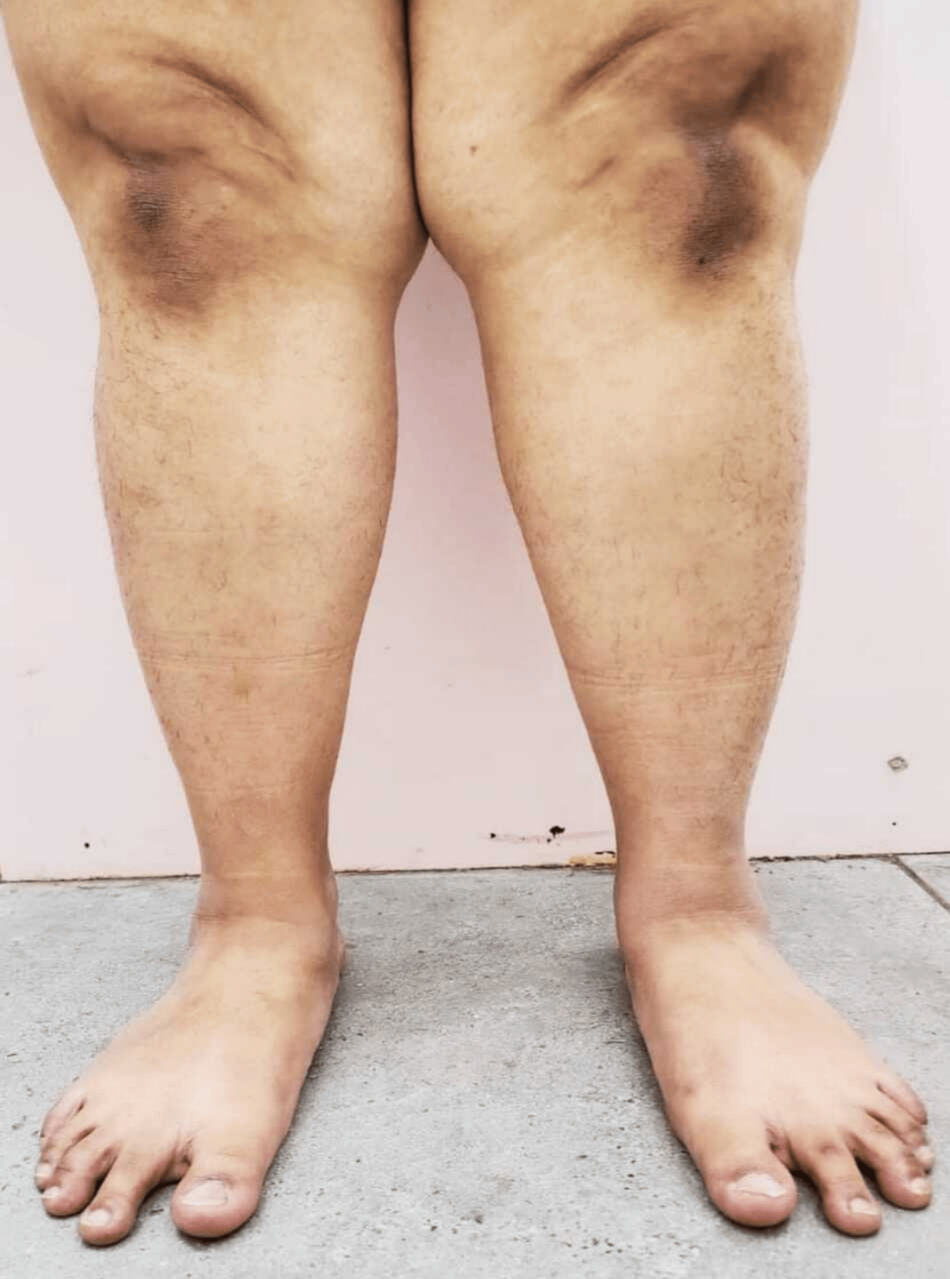
Anterior view of lower limb of a 23-year-old female showcasing the foot posture deformities including increase in external tibial torsion, knock knees and collapse of medial longitudinal arch of both feet

Figure 2 showcases an anterior view of the lower limb. It depicts the genu valgum or knock knees. It is the distance between the medial malleoli in a standing patient with touching medial femoral condyles.

**Figure 3:**
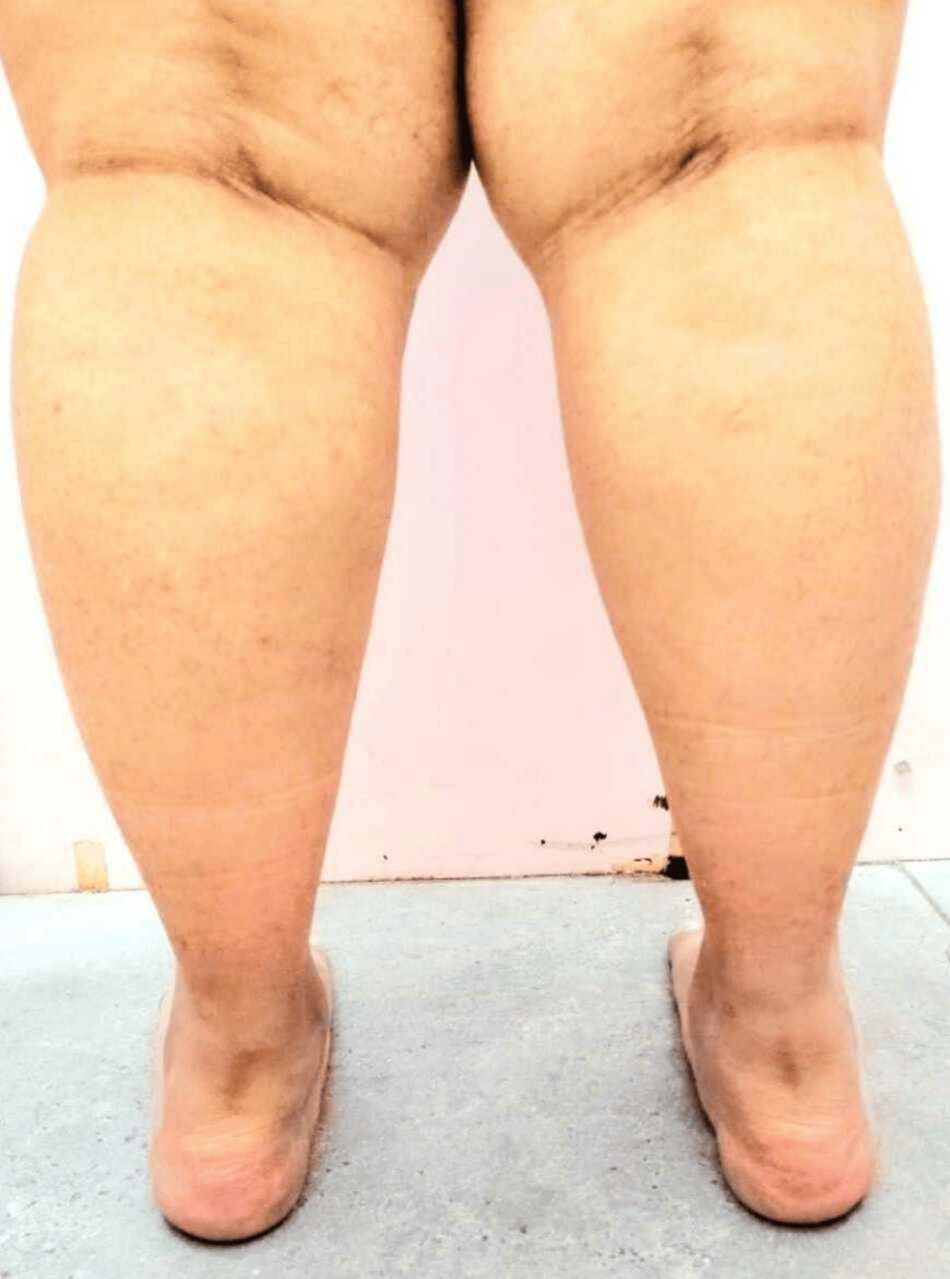
Posterior view of lower limb of a 23-year-old female showcasing the foot posture deformities including increased calcaneovalgus and rearfoot valgus in both feet

Figure 3 showcases a posterior view of the lower limb and illustrates the orientation of the calcaneus in relation to the perpendicular plane, highlighting its alignment and structural positioning. This depiction is essential for understanding foot mechanics and its role in maintaining balance and stability during various activities.

**Figure 4:**
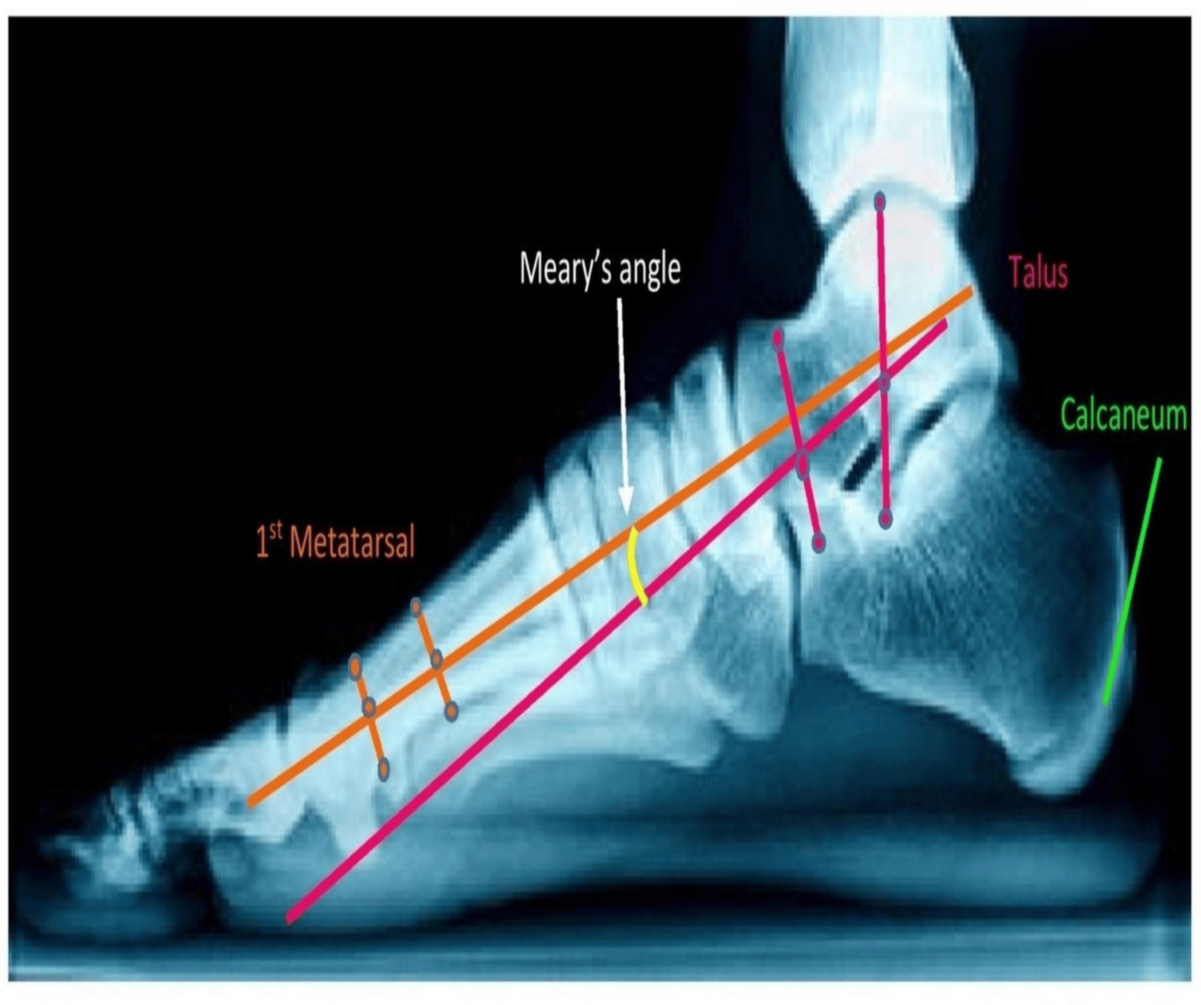
Meary's angle of the foot of a 41-year-old female which measures the tilting of both the talus and the first metatarsal bones

Figure 4 showcases regarding MA. It is a key radiographic measurement that helps evaluate the alignment of the foot, specifically the relationship between the hindfoot and forefoot. It measures the tilting of both the talus and the first metatarsal bones.

Statistical analysis 

The statistical analysis of the obtained data was conducted using IBM SPSS Statistics software version 26 (IBM Corp., Armonk, NY). The percentage of each outcome measure for each group was calculated. An unpaired t-test was used to compare the outcome measures between the two groups.

## Results

Table 1 provides a summary of the demographic distribution of participants in the research. It reveals that there were equal numbers of participants found in each group and each gender subdivision. There was a total of 25% in each age group category. Each gender sub-category has an aggregate of 50% of the total samples. This equality between both groups gives a better insight into making a better comparative analysis of both groups.

**Table 1:** Sociodemographic status of participants Normal BMI range: 18.5-24.9 kg/m^2^ BMI:body mass index

Demographic variables		Normal BMI group		Obese BMI group	
		Number of participants	% of participants	Number of participants	% of participants
Age group, years	18-26	27	25%	27	25%
	27-34	27	25%	27	25%
	35-42	27	25%	27	25%
	43-50	27	25%	27	25%
Gender	Male	54	50%	54	50%
	Female	54	50%	54	50%

Table 2 details the FPI-6 scores based on age and gender. In the obese BMI group, 20 (18.51%) participants from the age group 43-50 years showed planus deformity on the contrary, the normal BMI group showed a smaller number of planus deformities in the age group 43-50 years. This showed that the obese BMI group is prone to foot posture changes and elderly people had these changes in the obese BMI group than the normal group (p=0.0003). When comparing the gender of these two groups, 39 (36.11%) females of the obese BMI group were affected more than males underlying that females are prone to foot posture alterations than males (p=0.0002).

**Table 2:** Foot posture index assessment on the basis of age and gender Normal BMI range: 18.5-24.9 kg/m^2^. Normal FPI-6 score: 0 to +5 An unpaired t-test was used to check the statistical significance BMI: body mass index; FPI-6: Foot Posture Index-6

Age group (years) and gender	Obese BMI group		Normal BMI group		P-value
	Normal	Abnormal	Normal	Abnormal	
18-26	9 (8.33%)	18 (16.66)	15 (13.88%)	12 (11.11%)	0.2081
27-34	12 (11.11%)	15 (13.88)	20 (18.51%)	7 (6.48%)	0.0251
35-42	11 (10.18)	16 (14.8%)	24 (22.22%)	3 (2.77%)	0.0294
43-50	7 (6.48%)	20 (18.51%)	23 (21.29%)	4 (3.70%)	0.0003
Male	18 (16.66%)	36 (33.33%)	42 (38.88%)	12 (11.11%)	0.1072
Female	15 (13.88%)	39 (36.11%)	35 (32.40%)	19 (17.59%)	0.0002

Table 3 summarizes the prevalence of normal and abnormal navicular drop values. This shows a higher degree of foot pronation or flattening of the medial longitudinal arch when standing. This proportion reflects an obese subset of female participants may be significantly affected (p<0.0001) by foot posture abnormalities. Nineteen participants from the age group of 43-50 years and 39 females from the obese BMI group were affected.

**Table 3:** Navicular drop test assessment on the basis of age and gender Normal BMI range: 18.5-24.9 kg/m^2^. Normal navicular drop: 6-10 mm An unpaired t-test was used to check the statistical significance BMI: body mass index

Age group (years) and gender	Obese BMI group		Normal BMI group		P-value
	Normal	Abnormal	Normal	Abnormal	
18-26	22 (20.37%)	5 (4.62%)	25 (23.14)	2 (1.85%)	0.0733
27-34	19 (17.59%)	8 (7.40%)	24 (22.22%)	3 (2.77%)	0.0760
35-42	16 (14.81%)	11 (10.18%)	25 (23.14%)	2 (1.85%)	0.0072
43-50	8 (7.40%)	19 (17.59%)	22 (20.37%)	5 (4.62%)	0.0032
Male	12 (11.11%)	42 (38.88%)	46 (42.59%)	8 (7.40%)	0.2333
Female	15 (13.8%)	39 (36.11%)	42 (38.88%)	12 (11.11%)	0.0001

Table 4 analyzes foot progression angle by gender and age. Among males, very few had a normal toe-out angle. In females, a significant p-value of 0.0018 indicates a higher prevalence of abnormal angles, which can lead to gait abnormalities. Age-wise, the obese 43-50 age category displayed a larger amount of participants with abnormal toe-out with a remarkable p-value of 0.0007 which portrays an extremely excellent relation between the obese elderly more prone towards having a greater toe-out angle and further leading to gait complication.

**Table 4:** Toe-out angle on the basis of age and gender Normal BMI range: 18.5-24.9 kg/m^2^. Normal toe-out angle: 5-18^0^ An unpaired t-test was used to check the statistical significance BMI: body mass index

Age group (years) and gender	Obese BMI group		Normal BMI group		P-value
	Normal	Abnormal	Normal	Abnormal	
18-26	9 (8.33%)	18 (16.66%)	20 (18.51%)	7 (6.48%)	0.0156
27-34	20 (18.51%)	7 (6.48%)	25 (23.14%)	2 (1.85%)	0.0148
35-42	19 (17.59%)	8 (7.40%)	24 (22.22%)	3 (2.77%)	0.0045
43-50	7 (6.48%)	20 (18.51%)	17 (15.74%)	10 (9.25%)	0.0007
Male	40 (37.03%)	14 (12.96%)	46 (42.59%)	8 (7.40%)	0.1067
Female	19 (17.59%)	35 (32.40%)	33 (30.55%)	21 (19.44%)	0.0018

Table 5 details the forefoot and hindfoot postural deviations seen in both groups. In the obese BMI group, both hindfoot valgus and forefoot varus were seen prominently compared to the normal BMI group. In the older population and females, these deviations were significantly seen p<0.0001. So, along with flatfoot, these postural malalignments were present in the obese individuals.

**Table 5:** Forefoot and rearfoot deviations on the basis of age and gender Normal BMI range: 18.5-24.9 kg/m^2^. Normal hindfoot valgus angle: 2-6^0^. Normal forefoot varus angle: 1-8^0^ An unpaired t-test was used to check the statistical significance BMI: body mass index

Age group (years) and gender	Obese BMI group		Normal BMI group		P-value
	Abnormal hindfoot valgus + forefoot varus	None of these	Abnormal hindfoot valgus + forefoot varus	None of these	
18-26	25 (23.14%)	2 (1.85%)	7 (6.48%)	20 (18.51%)	0.0254
27-34	24 (22.22%)	3 (2.77%)	5 (4.62%)	22 (20.37%)	0.0007
35-42	26 (24.07%)	1 (0.92%)	5 (4.62%)	22 (20.37%)	<0.0001
43-50	26 (24.07%)	1 (0.92%)	9 (8.33%)	18 (16.66%)	<0.0001
Male	52 (48.14%)	2 (1.58%)	7 (6.48%)	47 (43.51%)	0.0078
Female	50 (46.29%)	4 (3.70%)	6 (5.55%)	48 (37.96%)	<0.0001

Table 6 provides a summary of MA in both groups. In the obese BMI group, the age group 35-42 and age group 43-50 years were significantly more affected than the normal BMI group. Also, when comparing the genders, both males and females were significantly affected in the obese BMI group than other groups.

**Table 6:** Meary’s angle according to age and gender Normal BMI range: 18.5-24.9 kg/m^2^. Normal Meary's angle: <4^0^ An unpaired t-test was used to check the statistical significance BMI: body mass index

Age group (years) and gender	Obese BMI group		Normal BMI group		P-value
	Affected	None	Affected	None	
18-26	25 (23.14%)	2 (1.85%)	5 (4.62%)	22 (20.37%)	0.0024
27-34	21 (19.44%)	6 (5.55%)	4 (3.70%)	23 (21.29%)	0.0476
35-42	26 (24.07%)	1 (0.92%)	4 (3.70%)	23 (21.29%)	<0.0001
43-50	25 (23.14%)	2 (1.85%)	7 (6.48%)	20 (18.51%)	<0.0001
Male	49 (45.37%)	5 (4.62%)	7 (6.48%)	47 (43.51%)	<0.0001
Female	52 (48.14%)	2 (1.85%)	5 (4.62%)	49 (45.37%)	<0.0001

## Discussion

In the present study, which involved 216 participants (108 males and females), we utilized the FPI-6, navicular drop test, foot progression angle, MA, forefoot, and rearfoot deviations to assess foot posture. Our findings revealed significant gender and age-related differences in foot posture. Females had greater affection than males. The elderly population especially the 43-50 age group affected significantly than other age groups. A previous study indicated that obese individuals experienced significantly higher levels of foot pain and reported lower scores on the SF-36 quality of life survey. Obesity was also associated with foot-related functional limitations, including reductions in ankle dorsiflexion strength, as well as diminished strength in the hallux and lesser toes, and decreased stride and step length, along with slower walking speeds when compared to individuals with lower body weight. Consequently, obesity in older adults leads to debilitating foot pain, structural changes in the foot, and impaired foot function, all of which negatively impact their overall quality of life [20].

In another study, obese participants exhibited flatter feet, a restricted range of motion in inversion-eversion, and increased peak plantar pressures during walking. After adjusting for foot structure and walking speed, body weight was found to significantly contribute to heightened foot loading, particularly in the forefoot and midfoot regions. These findings suggest that obesity increases mechanical stresses on the foot both directly, through excess body weight and indirectly, through alterations in foot structure [21]. A further investigation noted increased patterns of foot loading at the midfoot during walking in obese individuals, which may be attributable to an excess of adipose tissue in the medial arch. This observation supports the notion that it is the fat mass, rather than alterations in foot structure, that may contribute to elevated midfoot plantar pressures and the outward appearance of a "flat" foot in obese individuals [22].

In another study, both men and women with obesity were found to be more susceptible to foot pain compared to individuals of normal weight. Foot posture (normal, cavus, planus) and dynamic foot function (normal, supinated, pronated) were defined using a plantar pressure measurement system. In men, severe obesity was associated with an increased prevalence of foot pain, whereas in women, individuals with overweight, moderate obesity, and severe obesity were similarly more likely to experience foot pain [23].

It is evident from the research that foot structure and function are compromised in overweight and obese individuals, irrespective of age, with these changes manifesting as early as the preschool years. Obese individuals tend to have larger, broader, and flatter feet compared to their leaner counterparts, with the flattening of the foot structure likely caused by a combination of a decreased height of the medial longitudinal arch and a thicker midfoot plantar fat pad. These structural changes are accompanied by a higher prevalence of foot pain and increased dynamic plantar peak pressures in overweight and obese children, with these pressures progressing from the midfoot area in preschool-aged children to include the midfoot and forefoot in primary school children, and eventually affecting the midfoot, forefoot, and heel regions in obese adults, depending on the degree of obesity [24].

Güvener et al. conducted a study examining the relationship between talar head thickness and foot arch variations. Their research, using ultrasound imaging, found a clear association between increased talar head thickness and pes planus, particularly in obese individuals. These findings further underscore the role of obesity in the alteration of foot structure due to increased body mass [25]. Unver B et al. conducted a comprehensive review investigating the relationship between foot posture, muscle strength, range of motion, and plantar sensation in obese and overweight individuals. Their study revealed that obese individuals had higher FPI scores than their overweight counterparts, suggesting that obesity significantly impacts foot posture [26].

Overall, our study provides deeper insights into the impact of obesity on foot health and underscores the need for ongoing research in this area. Through comprehensive assessments of foot posture, healthcare professionals can design interventions that specifically address the challenges faced by this population, ultimately leading to improved outcomes and enhanced quality of life. Moreover, the study underscores the importance of preventive strategies. Timely detection and intervention can mitigate the progression of foot posture disorders, thereby reducing the risk of more severe complications in the future.

Strengths

This study offers valuable insights into the various aspects of foot malalignments in individuals with obesity. By highlighting the association between obesity and foot posture abnormalities, the research serves as an essential tool for early diagnosis and intervention. Early identification of these malalignments enables the implementation of targeted interventions that can improve prognosis and prevent the development of further complications. The findings of this research will inform the creation of reliable and effective rehabilitation plans tailored to the unique needs of obese individuals.

Limitations

The study provides data from a single time point, which limits its ability to assess the long-term assessments and follow-up of the participants. The study primarily utilized the Foot Posture Index, Navicular Drop Test, degree of toe-out evaluation, forefoot and rearfoot deviations, and MA which provided valuable insights. However, incorporating advanced assessment tools, such as 3D foot scanners, accelerometers, inertial measurement units, and force plate analysis, could have provided more precise and objective measurements of foot posture malalignments and gait-related abnormalities caused due to it.

Recommendations

This study provides a foundation for further research by identifying several areas that warrant deeper investigation. One critical area for future exploration is the assessment of fat mass and its correlation with foot malalignments and gait abnormalities. Further research into how varying levels of fat mass impact foot posture and function could enhance our understanding of the relationship between obesity and foot health. Additionally, incorporating pain assessments during walking activities could offer important insights into the impact of foot malalignments on daily functional capabilities and overall quality of life. Integrating pain measurements into gait analysis may uncover the extent to which discomfort influences biomechanical movement patterns and mobility. Overall, this research emphasizes the need for a detailed, individualized approach to managing foot malalignments in obese individuals.

## Conclusions

This study aimed to evaluate foot malalignments in the obese population, with a focus on identifying prevalent abnormalities in foot posture. The results reveal that pes planus (flat feet) represents the most common foot posture deviation, particularly among females and the older population along with abnormal deviations in forefoot varus and hindfoot valgus. Also, affected FPI-6, navicular drop, degree of toe out, and MA in the obese BMI group highlighted foot posture abnormalities. Early screening enables timely interventions, such as orthotic support, physiotherapy, or lifestyle modifications, which may help prevent further complications. Additionally, weight loss is generally associated with reduced musculoskeletal strain, which could potentially alleviate symptoms and improve function. These findings contribute to a more nuanced understanding of foot biomechanics in individuals with obesity, offering valuable insights into the functional dynamics of foot alignment and its broader implications on overall movement.
